# Prospective Clinical Evaluation of Posterior Third-Generation Monolithic Zirconia Crowns Fabricated with Complete Digital Workflow: Two-Year Follow-Up

**DOI:** 10.3390/ma15020672

**Published:** 2022-01-17

**Authors:** Mustafa Gseibat, Pablo Sevilla, Carlos Lopez-Suarez, Verónica Rodríguez, Jesús Peláez, María J. Suárez

**Affiliations:** Department of Conservative Dentistry and Buccofacial Prostheses, Faculty of Odontology, University Complutense of Madrid, 28040 Madrid, Spain; mam@ucm.es (M.G.); pasevi01@ucm.es (P.S.); carlop04@ucm.es (C.L.-S.); veranicr@ucm.es (V.R.); mjsuarez@ucm.es (M.J.S.)

**Keywords:** single crown, zirconia, posterior crown, full-coverage restoration, veneered zirconia, monolithic zirconia, yttria-stabilized tetragonal zirconia

## Abstract

Clinical studies on the behavior of posterior translucent monolithic zirconia restorations are lacking. We assessed the clinical outcome and survival rate of posterior third-generation monolithic zirconia crowns over a 2-year period. A total of 24 patients, requiring 30 posterior full-contour restorations were selected. All abutments were scanned, and crowns were milled and cemented with a self-adhesive dual cure cement. Crowns were assessed using the California Dental Association’s criteria. Gingival status was assessed by evaluating the gingival index, plaque index, periodontal probing depth of the abutments and control teeth, and the margin index of the abutment teeth. Statistical analyses were performed using the Friedman and the Wilcoxon signed-rank tests. During the 2-year follow-up, no biological or mechanical complications were observed, and the survival and success rate was 100%. All restorations ranked as satisfactory throughout the follow-up period. The gingival index and plaque index were worse at the end of the 2-year follow-up. The margin index was stable during the 2 years of clinical service. No significant differences were recorded in periodontal parameters between crowns and control teeth. Third-generation monolithic zirconia could be a reliable alternative to posterior metal–ceramic and second-generation monolithic zirconia posterior crowns.

## 1. Introduction

Metal–ceramic restorations are still considered the “gold standard” for the rehabilitation of posterior teeth due to their long-term predictability. However, the increasing demand for aesthetic restorations has led to an increase in the use of dental ceramics in the posterior region [1,2]. Conventional ceramics have limited use due to their low flexural strength, a shortcoming that caused researchers in recent years to direct their work toward the development of new materials and techniques to increase their resistance to fracture.

Zirconium-oxide-based ceramic systems have been introduced in recent years. It is the most resistant and stable ceramic material, with great mechanical and biocompatibility properties [1,2,3]. However, zirconia has an opaque structure with low translucency, and must be veneered by felspathic ceramic to improve the restoration’s aesthetic appearance [4,5]. Layered zirconia has shown good clinical results compared with metal–ceramic restorations. However, despite its high survival rate (>90%) and adequate aesthetics, the chipping or fracture of the veneering ceramic (16.97%) after 10 years of follow-up is a common complication, and a cause of failure of this type of zirconia restorations [2,6,7,8,9,10]. The core–ceramic veneering interface is one of the weakest aspects of the layered zirconia [1,2,4,5]. In order to overcome this weakness, monolithic zirconia has been introduced [11,12,13]. Therefore, in recent years, there has been a growing interest in the use of monolithic zirconia restorations. Monolithic zirconia shows a satisfactory aesthetic, requires more conservative dental preparation, less laboratory time, and fewer clinical sessions, and since it is monolithic, it lacks the unwanted complication of chipping [11,13,14]. The latest modifications in zirconia composition, structure, and fabrication methods have resulted in translucent monolithic zirconia (the third generation), but with a significant reduction in the flexural strength [15,16]. However, due to their recent introduction, studies on their clinical outcome are still limited, and most of them are in vitro. Clinical studies are still very scarce [17,18], and results are not conclusive. Therefore, more clinical studies are necessary to evaluate their clinical behaviour.

Three generations of zirconia have been introduced to the market. The first generation, also known as “conventional zirconia”, is mainly used to manufacture the core of ceramic crowns and fixed prostheses [6,15,19]. It must be veneered by feldspathic ceramic to enhance the aesthetics. In addition, it is also used as an alternative to implants and titanium abutments [14,19,20]. Zirconia implants seem to be a promising alternative to titanium due to their superior soft-tissue response, biocompatibility, and comparable osseointegration. Nevertheless, the titanium implants exhibited superior mechanical properties [21,22]. The first generation of prosthetic indications is characterized by its high flexural strength (1000 to 1500 MPa), biocompatibility, good integrity to soft tissues, high sintering temperature (1600 °C), and long working time [6,15,19,20]. However, its main complication is the chipping of the veneering ceramic [1,2,4,13,23]. In 2011, the second-generation zirconia was introduced. The reason for its introduction was to improve the translucency of the first generation without changing the mechanical properties. By reducing the concentration of the additive alumina and eliminating the porosity by sintering at a higher temperature, a more translucent zirconia than the previous generation was achieved [6,15,19]. However, this generation was not yet aesthetic enough to be used in aesthetic areas or as monolithic restorations in the anterior zone [15,19,24]. However, it remains suitable for fabricating posterior monolithic restorations. This generation exhibited an acceptable translucency and high flexural strength (900 to 1300 MPa) [19]. As a result of the continuing development of zirconia, the third generation was launched in 2015 [15,19,24], to further reduce the opacity, and to achieve an aesthetic result comparable to the other types of ceramics. Conversely, when compared with the first and second generation, it is not only stable in the tetragonal phase, but it also contains a proportion of a cubic phase of up to 53%. It can be described as partially stabilized zirconia (PSZ) [19], when the C phase is less than 50%, and it can be described as fully stabilized zirconia (FSZ) [6] when the C phase is more than 50%. Cubic crystals are larger in size compared with tetragonal crystals, and this means that the light is scattered less strongly at the crystal boundaries and residual porosities, making the material more translucent [6,19,25,26]. On the other hand, the flexural strength significantly decreases (400 to 1000 MPa) [15,19,26].

The aim of this prospective clinical trial was to evaluate the clinical outcome and survival of posterior third-generation monolithic zirconia crowns. The null hypothesis was that no differences would be found from baseline to 3-year follow-up among the assessed parameters.

## 2. Materials and Methods

### 2.1. Patient Selection

The present study was conducted in conformity with the Declaration of Helsinki. The design of this prospective clinical trial was approved by the Ethical Committee of Clinical Trial at San Carlos University Clinical Hospital (Madrid, Spain) (C.P.- C.I. 19/002-E). The study was registered in ClinicalTrials.gov (Identifier NCT 04943315). The patients were selected from among those who attended the dental clinic of the Master in Bucofacial Prosthesis and Occlusion (Faculty of Odontology, University Complutense of Madrid, Spain—UCM) from January 2019 to March 2019, in whom the realization of at least one total coverage restoration in the posterior maxillary or mandibular regions was indicated. A total of 39 patients were screened and examined. Of those 39 patients screened, 24 patients (7 males and 17 females) in need of 30 single posterior crowns fulfilled the inclusion criteria and were enrolled in the study. No power analysis was performed, and the sample size was determined according to previous studies [2,27,28,29,30,31].

The inclusion criteria were as follows: patients over the age of 18 years who present a posterior tooth (molar or premolar) in need of a total coverage crown, vital abutment or adequate endodontic treatment, abutment not previously crowned, periodontally healthy abutment without signs of bone resorption or periapical lesion, adequate occluso-gingival height (4 mm or more), favorable crown/root ratio, and stable occlusion. Patients with poor oral hygiene, signs of bruxism, or who refused the follow-up appointments were excluded. The clinical phase of the treatment was carried out by two students in the 3rd year of the Master in Bucofacial Prosthesis and Occlusion of the UCM, with experience in the use of zirconia restorations and the intraoral scanner (IOS). Patients received oral hygiene instructions and signed an informed consent prior to the treatment.

### 2.2. Clinical Procedures

**Figure 1:**
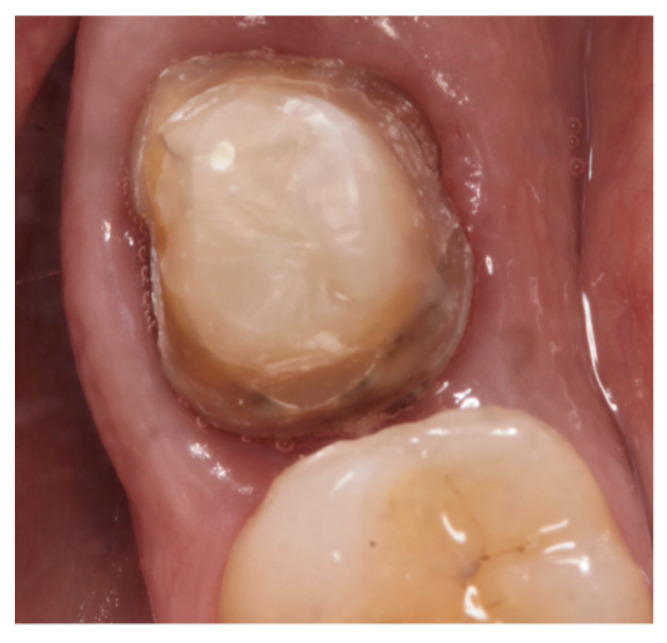
Abutment preparation.

**Figure 2:**
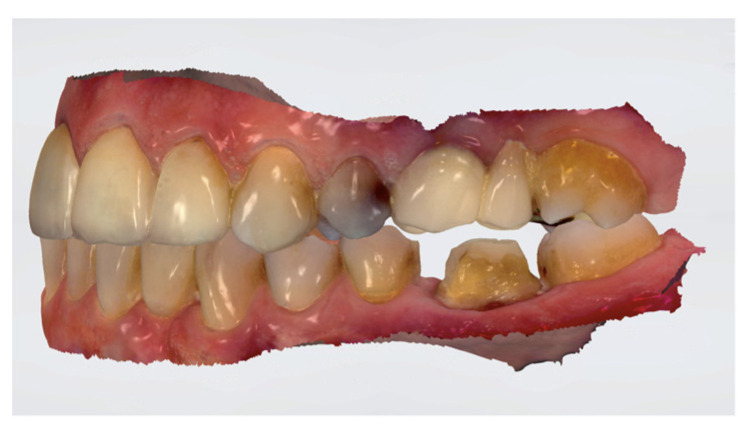
Digital impression.

Dental preparations were made following the manufacturer’s recommendations. The abutments were prepared as follows: isogingival chamfer finish line, axial reduction of approximately 1 mm, and occlusal reduction of approximately 1 mm without any sharp angles. The convergence between axial walls was approximately ≤6° degrees (Figure 1). Impressions and occlusal bite were taken using an IOS (Trios 3: 3Shape, Copenhagen, Denmark) (Figure 2), with the use of a lip and cheek retractor (OptraGate; Ivoclar Vivadent, Schaan, Liechtenstein), and a retraction cord (Ultrapak E; Ultradent, South Jordan, UT, USA). Temporary restorations (Protemp Crown; 3M ESPE, Seefeld, Germany) were made and cemented using a temporary cement (RelyX tem; 3M ESPE). The shade was selected using a digital spectrophotometer (VITA Easyshade V; Vita Zahnfabrik, Essen, Germany). All data of each patient were sent to the laboratory.

**Figure 3:**
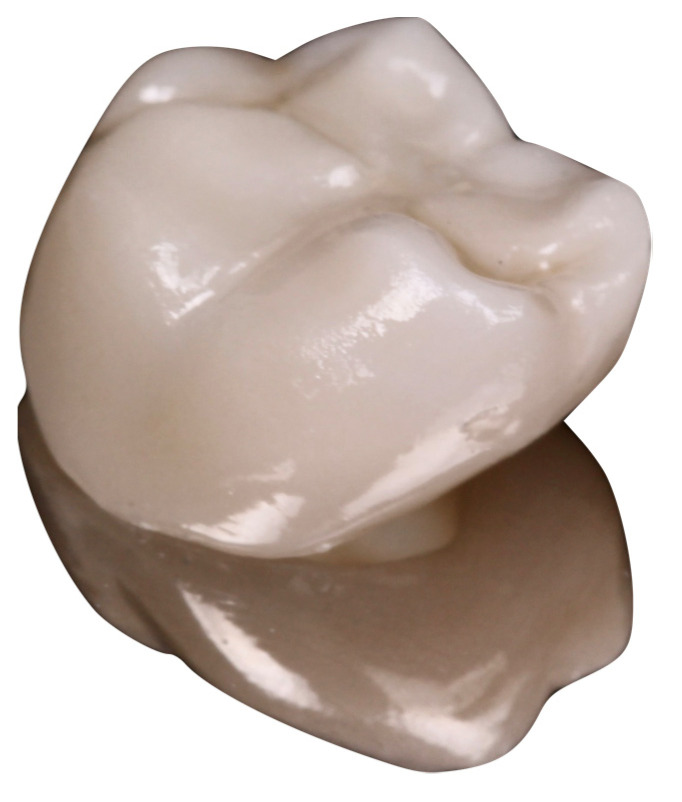
Finished MZC.

All restoration designs were carried out using the 3Shape software (3Shape) according to the properties of the material, anatomical limitations, and aesthetic expectations. Through the Wieland milled machine (Zenotec select hybrid; Wieland Dental, Pforzheim, Germany), all monolithic zirconia crowns (MZCs) were made from partially sintered super-translucent multilayered zirconia blanks (KATANA Zirconia STMAL; Kuraray Noritake, Miyoshi, Japan). This material combines a high degree of transparency with a good flexural strength (600–800 MPa). All restorations were sintered at 1.550 °C for 2 h in a furnace (Programat CS4; Ivoclar Vivadent). The sintered restorations were characterized and glazed with Cerabien ZR (Kuraray Noritake) at 750 °C for 10 min, following the manufacturer’s specifications in all producing steps (Figure 3). All crowns were prepared by an experienced technician.

**Figure 4:**
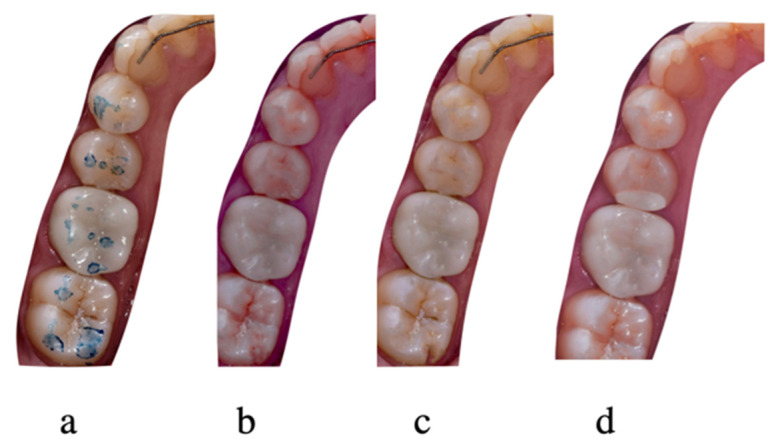
MZC evaluated at 1 week (**a**), 6 months (**b**), 1 year (**c**) and 2 years (**d**).

Prior to the cementation step, marginal fit, occlusion, shade, contact points, and patient satisfaction for every restoration were checked. Once checked, the internal surface of the restorations was treated with 50 μm alumina particles at a pressure of 1 bar for 5 s at approximately 10 mm (CoJet; 3M ESPE), and was ultrasonically cleaned. The crowns were then cemented with a dual-cure, self-adhesive resin cement (Panavia SA Cement Universal; Kuraray Noritake) following the manufacturer’s protocol. After cementation, occlusal contacts were evaluated (Figure 4), and adjustments were made when necessary to ensure adequate contacts in maximum intercuspation without interference in laterotrusion movements. Adjusted surfaces were polished using a zirconia polishing kit (KATANA Zirconia Twist Dia; Kuraray Noritake).

### 2.3. Follow-Up Examination and Statistical Analysis

The restorations were assessed using the California Dental Association’s system (CDA), which evaluates the surface and color, anatomic form, and marginal integrity [2,27,30,31,32,33,34,35]. Every parameter was rated as satisfactory: excellent/acceptable, or non-satisfactory: repair/substitution [2,27,30,31,32,33,34,35]. The periodontal conditions were evaluated by recording the gingival index (GI), plaque index (PI), margin index (MI) and probing pocket depth (PPD) of the abutments and the corresponding contralateral teeth (control) [2,29,30,31,36]. All MZCs were evaluated at 1 week (baseline), 6 months, 1 year and 2 years by 2 calibrated investigators who were experienced in using the CDA quality assessment system, and who were not involved in the restorative treatment. Each evaluator evaluated the MZCs and the control teeth independently, and the worst result was used in the case of discrepancies. MZCs with “excellent” or “acceptable” rates were considered successful, and MZCs with “repair” or “substitution” rates were considered failed. Survival was defined as the restoration remaining in situ over the entire follow-up period [4,37].

Data analysis was performed with statistical software (IBM SPSS Statistics v25.0; IBM Corp, ArmonK, NY, USA). Descriptive statistics were applied for the evaluation of the restorations and the control teeth outcomes. The Friedman test was applied for the comparisons of the baseline and the follow-up values. The Wilcoxon signed-rank test was applied to compare the differences in periodontal parameters between abutments and control teeth. It was also used for matched pairs on MZCs to evaluate differences considering the periodontal parameters and the CDA criteria. In order to be able to handle the data referring to the CDA criteria more easily, they were given a numerical value on a scale from 1 to 4, where 4 = excellent, 3 = acceptable, 2 = repair, and 1 = substitution. The periodontal parameters were catalogued by assigning a score from 0 to 3 (PI and GI) or from 1 to 4 (MI and PPD). The level of significance was established at α = 0.05.

## 3. Results

A total of 30 MZCs were placed in 24 patients (17 females, 7 males) with a mean age of 55.3 ± 13.9 years. The restorations distribution is shown in Table 1, and all participants were evaluated for 24 months. The survival and success rate were 100% at 2 years, and no mechanical or biological complications were recorded. All restorations were ranked as satisfactory after 2 years of clinical service.

The recorded CDA ratings at each follow-up evaluation are listed in Table 2. Surface and color, anatomic form and marginal integrity did not show any significant differences when the 6-month data, 1-year data and 2-year data were compared with the baseline data. In terms of marginal integrity, all MZCs (100%) were scored as excellent at all follow-up periods.

The periodontal parameters scores are shown in Table 3 and Table 4. The GI was significantly higher at 6 months (*p* = 0.025), 1 year (*p* = 0.008), and at the 2-year follow-up (*p* = 0.02) when compared with the baseline. The GI at 6 months did not differ significantly when compared with the GI at 1 year and at 2 years (*p* = 0.02). The PI was worse at 2-year follow-up than at baseline. Significant differences were recorded when the PI at baseline was compared with the PI at 6 months (*p* = 0.046), 1 year (*p* = 0.011), and 2 years (*p* = 0.004). Regarding the PPD, no significant differences were observed when the baseline and the follow-up values were compared. At 2-year follow-up, most of the MZCs (93.6%) had a PPD in the range of 1 to 3 mm. With respect to the MI, it was stable in all follow-up evaluations in all MSCs. The comparison between the test and the control teeth did not show any significant differences for any of the periodontal parameters.

## 4. Discussion

The result of the statistical analysis of this study supports the partial rejection of the null hypothesis, as differences were detected for periodontal parameters among the follow-up periods.

This study evaluated the survival, success, and complications of third-generation posterior monolithic zirconia crowns for 2 years. Twenty-four patients (30 crowns) were included in the study and no participant failed to attend during the time of observation.

Due to the increasing aesthetic demands by patients, and to overcome the aesthetic limitations of metal–ceramic restorations, all-ceramic restorations have been introduced [5]. The use of zirconia in the last decade has grown greatly. At the beginning, it was used as a core for single restorations and fixed partial prostheses for its optimal mechanical and aesthetic properties, biocompatibility, and low thermal conductivity [4,5,6,7,8,9,10]. However, many studies concluded that the fracture of the veneering ceramic (chipping) was the most frequent complication of bilayered zirconia [7,11,12,13,14,15]. There are several possible causes of chipping: compression forces and thermal expansion resulting from the sintering process, the differences in the modulus of elasticity between the core and the veneer material, the bonding between the core and the veneer ceramic, improper ceramic thickness, and malocclusion [12,17,18,19]. To overcome this mechanical limitation, monolithic zirconia was introduced [11,15,20,23,24,25,26].

In the present study, a recently introduced third-generation zirconia was used, so previous clinical studies are very scarce. Most of the previous studies evaluated the second-generation zirconia, reporting survival rates from 1 to 5 years of follow-up in the range of 86.7% to 100%, which is consistent with the results of the present study [29,31,38,39,40,41,42,43,44,45]. A systematic review of the survival, complications, and clinical behaviour of monolithic tooth-supported zirconia crowns reported a 1-year survival rate ranging from 91% to 100% [46]. In addition, most of the studies were based on a short period of follow-up with a small number of restorations. Koenig et al. [28], performed a prospective study with two years of clinical follow-up, including single tooth, implant-supported crowns, and posterior partial fixed prostheses of second-generation monolithic zirconia (Lava Plus 3M ESPE). They reported a survival rate of 76.9% for tooth-supported crowns, obtaining a clinical evaluation ranging from “acceptable” to “excellent” that was not consistent with the results of this study. Moreover, in the same study, the authors included patients with signs of bruxism and the mechanical complications that have been linked to bruxism. The present study did not include any patient with signs of bruxism. However, in order to be able to talk about reliable results, a longer follow-up period is necessary.

To the best of the authors knowledge, only two studies have evaluated the third-generation zirconia [17,18]. Pathan et al. [17], performed a prospective one-year clinical follow-up study evaluating tooth-supported posterior third-generation monolithic zirconia crowns (DG Star, DentGallop, Huston, TX, USA). They reported a survival rate of 100% (60 crowns). All crowns had an “excellent” clinical evaluation, which is consistent with the results of this study. Worni et al. [18], published a case series of tooth-supported crowns and fixed partial prostheses fabricated from a third-generation monolithic zirconia (Ceramill Zolid FX, Amann Girrbach AG, Koblach, Austria) with 3 years of clinical follow-up. They reported a survival rate of 100% and all restorations obtained a clinical evaluation of “acceptable”, which is consistent with this current study.

In the present study, no mechanical complications were observed, and no fracture or crack was recorded in any crown during the 2 years of clinical service, nor was any type of biological complications, secondary caries, pulpitis, or hypersensitivity recorded. These results are consistent with those obtained in previous studies. [28,29,31,39,41,47,48]. However, other authors detected some mechanical and biological complications such as debonding, root fracture, crown fracture, abutment fracture, cracks, endodontic problems, pulpitis, and secondary caries [40,42,43]. In the literature, some authors observed a number of complications in the opposing teeth such as root fracture, veneering ceramic fracture, composite fracture, and cracks and enamel cracks [49]. Suliman et al. [50], published a dental laboratory survey with the objective of determining the failure rate of monolithic zirconia restorations due to fractures after 5 years of clinical functioning. All systems evaluated in the study were second-generation zirconia, and a 0.69% fracture rate was estimated for posterior monolithic zirconia crowns at 5 years of clinical follow-up.

The marginal fit results obtained in the present study are satisfactory from the clinical point of view, and consistent with those obtained in previous studies [28,29,31,39,40,41,48,49,51,52,53,54,55]. No previous studies reported inadequate results at the level of marginal fit. Studies, both in vitro and in vivo, have shown good results of monolithic zirconia at the level of marginal fit. In vitro studies reported that monolithic zirconia crowns showed a clinically acceptable marginal fit, and better marginal fit than veneered zirconia crowns and metal–ceramic crowns [56,57,58,59]. It also suggested that the rounded shoulder finish line or deep chamfer may have an optimal geometry to minimize occlusal stress [9]. The clinical studies published by Rau et al. [60] and Batson et al. [61] concluded that the monolithic zirconia crowns showed a clinically acceptable occlusal and marginal fit. In another clinical study conducted by Sakornmiman et al. [62], no significant differences between monolithic zirconia crowns fabricated using intraoral digital impressions and those fabricated using conventional silicone impressions at the marginal fit level were reported.

The CDA system for the evaluation of the quality of restorations is a useful instrument, but it uses a subjective criterion, and in longitudinal studies there may be a risk of changes in the decision levels from one observation period to another. In this study, an effort was made to standardize the evaluation method. In this study, the CDA rating of satisfactory was granted for 100% of all crowns in all evaluation periods after 2 years of clinical follow-up. All obtained results in the evaluation of the surface and color, the anatomical form, and the marginal integrity were excellent or acceptable. These results were similar to those obtained by most previous studies [28,29,31,39,40,41]. In the current study, after 2 years of clinical service, most of the crowns (86.7%) obtained an excellent evaluation in the four evaluation periods, and only two crowns (13.3%) obtained an acceptable evaluation. In relation to the color and surface parameter, two crowns obtained an acceptable rating at baseline evaluation due to a minimal discrepancy in translucency. However, this slight variation in color was detected at the cementation appointment, and despite this mismatch, the patients were very satisfied. Two crowns were dropped from excellent to acceptable rating, both of them due to a slight wear and a loss of translucency at the occlusal surface, which is consistent with previous studies [28,29,31,39]. Regarding the anatomical form, a change from excellent to acceptable rating occurred in three crowns due to wear in the occlusal surfaces. However, no differences were observed after 2 years of function, which is consistent with previous studies [28]. During the 2-year follow-up, the soft tissue response was good. There were no significant differences between the crowns and the control teeth in the periodontal parameters. These results are consistent with those obtained in previous studies [27,28,61]. Throughout the follow-up period, a slight increase in PI and GI was observed in the crowns and control teeth, making the results slightly worse after the 2year follow-up with respect to the baseline, and consistent with those obtained in previous studies [18,28,36,61]. These results may be due to multiple factors, such as the patient’s hygiene habits, the patient’s own bacterial flora, periodontal maintenance, marginal adaptation, surface texture, and cement [30]. The PPD around the abutment teeth was stable in most cases (96.7%) during the 2-year follow-up. Throughout the follow-up period, no change in the location of the margin was observed in any restoration, which is consistent with the results obtained by Batson et al. [61], Koenig et al. [28] and Worni et al. [18]. These results may be due to the good marginal fit of the crowns, the biocompatibility of zirconium oxide, and good hygiene control [31,33,61].

In this study, a resin-based cement was used. During the observation period, no cases of debonding or abutment sensitivity were observed. The cementation protocol recommended by the manufacturer and by previous studies was followed [63,64,65,66,67,68]. Nakamura et al. [68] studied in vitro the effect of the cement type on the resistance to fracture of MZCs, and concluded that MZCs with a minimum thickness of 0.5 mm could have a good resistance to fracture regardless of the type of the cement. Another in vitro study concluded that the cement did not have a significant effect on stress distribution in MZCs [69]. According to Bayindir and Koseoglu [70], the thickness of the restoration and the color of the cement affect the final color and translucency of MZCs. Until the date of this study, there were no long-term clinical data evaluating the use of resin-based cements with MZCs.

In the presented study, a digital workflow was performed to ensure the maximum precision and marginal accuracy. The previous study demonstrated differences among different IOSs in terms of marginal discrepancy; however, all the IOSs tested showed clinically acceptable marginal discrepancies, and the authors concluded that the reliability of the workflow was enhanced by the advantages of the full digital environment [71]. Furthermore, temporary restorations were performed before zirconia crowns were carefully manufactured for aesthetic, functional and biologic reasons, and to allow the patients adequate hygiene [72].

The main limitation of this study was the sample size, which was small. A larger sample size would allow obtaining results with greater statistical relevance. In addition, the follow-up time was short, and it did not allow for evaluating the correct clinical behavior of this generation of MZCs. A longer observational period is required to validate these results. Furthermore, in the study, only the clinical behavior of translucent zirconia was analyzed, and there was not a control group.

## 5. Conclusions

The survival and success rate at 2 years were 100%, with no mechanical or biological complications. All restorations were ranked as satisfactory at 2-year follow-up. The excellent results obtained in this study suggest that the third-generation tooth-supported monolithic zirconia crowns in posterior regions seem to be a good alternative to metal–ceramic crowns, second-generation monolithic zirconia crowns, and veneered zirconia crowns. A long-term study is necessary to confirm this short-period study.

## Figures and Tables

**Table 1:** Distribution of restorations.

Tooth	Number of Restorations
Upper first premolar	3
Upper second premolar	7
Upper first molar	7
Upper second premolar	3
Lower first premolar	2
Lower second premolar	3
Lower first molar	4
Lower second molar	1

**Table 2:** Frequency (%) (number) of CDA evaluations at different follow-up periods.

CDA Assessment	Score	Baseline	6 Months	1 Year	2 Years
Surface and Color	4	93.3% (28)	90% (27)	86.7% (26)	86.7% (26)
	3	6.7% (2)	10% (3)	13.3% (4)	13.3% (4)
	2	0	0	0	0
	1	0	0	0	0
Anatomic Form	4	100% (30)	96.7% (29)	90% (27)	90% (27)
	3	0	3.3% (1)	10% (3)	10% (3)
	2	0	0	0	0
	1	0	0	0	0
Marginal Integrity	4	100% (30)	100% (30)	100% (30)	100% (30)
	3	0	0	0	0
	2	0	0	0	0
	1	0	0	0	0

**Table 3:** Gingival index (GI) (%) scores.

Gingival Index	Base Line		6 Months		1 Year		2 Years	
	Test	Control	Test	Control	Test	Control	Test	Control
0	96.7% (29)	96.7% (29)	80% (24)	76.7% (23)	70% (21)	70% (21)	70% (21)	66% (20)
1	0	3.3% (1)	16.7% (5)	20% (6)	20% (6)	26.7% (8)	30% (9)	30% (9)
2	3.3% (1)	0	3.3% (1)	3.3 (1)	10% (3)	3.3% (1)	0	3.3% (1)
3	0	0	0	0	0	0	0	0

**Table 4:** Plaque index (PI) (%) scores.

Plaque Index	Base Line		6 Months		1 Year		2 Years	
	Test	Control	Test	Control	Test	Control	Test	Control
0	96.7% (29)	70% (27)	83.3% (25)	76.7% (23)	66.7% (20)	66.7% (20)	60% (18)	56.7% (17)
1	0	30% (3)	13.3% (4)	20% (6)	33.3% (10)	30% (9)	40% (12)	43.3% (13)
2	3.3% (1)	0	3.3% (1)	3.3 (1)	0	3.3% (1)	0	3.3%
3	0	0	0	0	0	0	0	0

## Data Availability

No new data were created or analyzed in this study. Data sharing is not applicable to this article.
